# Neuromyelitis Optica Spectrum Disorders (NMOSDs) Diagnosed After Surgery for the Ossification of the Posterior Longitudinal Ligament of the Cervical and Thoracic Spine: A Case Report

**DOI:** 10.7759/cureus.61651

**Published:** 2024-06-04

**Authors:** Hiroyasu Kodama, Naohiro Kawamura, Satoshi Nagatani, Yuki Ishikawa, Junichi Kunogi

**Affiliations:** 1 Department of Spine and Orthopedic Surgery, Japanese Red Cross Medical Center, Tokyo, JPN

**Keywords:** aqp4-antibody, nmosd, opll, surgery, diagnosis

## Abstract

Complications of compressive spinal cord myelopathy and demyelinating disease can be difficult to diagnose. A 65-year-old woman gradually lost the ability to walk. Her imaging findings showed multiple spinal canal stenosis and ossification of the posterior longitudinal ligament in the cervical and thoracic spine. Some intramedullary signal changes were seen at sites distant from the spinal cord compression site. Although she underwent cervical and thoracic decompression and fusion surgery relatively early, her lower-extremity strength decreased after surgery. Her aquaporin 4 (AQP4)-antibody was found to be positive postoperatively, and she was diagnosed with NMOSD. Medical treatment for NMOSD improved her walking ability, and she finally became able to walk with a cane. In cases where there is a discrepancy between the site of strong stenosis and intramedullary signal changes, it is necessary to consider an anti-AQP4 antibody test and consultation with a neurologist.

## Introduction

Ossification of the posterior longitudinal ligament (OPLL) is a multifactorial disease caused by the interaction of genetic and environmental factors and can cause spinal cord compression. Surgical intervention should be considered for patients with neurologic symptoms, such as myelopathy or radiculopathy, and evidence of spinal cord compression [1].

In Japan, the incidence of OPLL among individuals presenting for evaluation with spinal disorders is between 1.9% and 4.3%, and in other Asian countries, the incidence is similarly reported up to 3.0% [2,3]. Conversely, a much lower prevalence of 0.1 to 1.7% is described in comparable North American and European cohorts [4-6]. OPLL is diagnosed when there is imaging evidence of ossification leading to spinal cord compression, accompanied by symptoms of myelopathy, such as motor dysfunction, sensory dysfunction, and bladder or bowel dysfunction, and a causal relationship is demonstrated between these symptoms and ligament ossification.

Neuromyelitis optica (NMO) is a chronic inflammatory autoimmune disease of the central nervous system (CNS) associated with a characteristic pattern of astrocyte dysfunction and loss, resulting in secondary demyelination and neurodegeneration [7]. In 2004, the discovery of a pathogenic NMO-associated IgG antibody, targeting the water channel membrane protein aquaporin 4, was an important milestone in differentiating NMO from multiple sclerosis (MS). After varying forms of clinical presentation were described for the disease, the term NMO spectrum disorder (NMOSD) was introduced in 2007 and now commonly used. Ocular pain with loss of vision and myelitis with severe symmetric paraplegia, sensory loss below the lesion, and bladder dysfunction are typical features of neuromyelitis optica [8]. The diagnostic criteria for AQP4 antibody-positive cases are as follows: The patient must have at least one core clinical feature, which may include optic neuritis, acute myelitis, area postrema syndrome (episodes of otherwise unexplained hiccups, nausea, or vomiting), acute brainstem syndrome, symptomatic narcolepsy or acute diencephalic clinical syndrome with NMOSD-typical diencephalic MRI lesions, or symptomatic cerebral syndrome with NMOSD-typical brain lesions. NMOSD is relatively common in non-White and populations with a minor European contribution to their genetic composition, such as AfroBrazilians (15% of cases of demyelinating disease), West Indians (27%), Japanese (20-30%), and east Asians, including Hong Kong Chinese (36%), Singaporeans (48%), and Indians (10-23%) [8]. 

MS, which is also a demyelinating disease, shows sensory changes, motor deficits, gait imbalance, and bowel or bladder dysfunction [9]. Many of these symptoms are also common in patients with cervical spondylotic myelopathy (CSM). The coincidence of CSM with demyelinating disorders has been reported in the past, and diagnosis and treatment tend to be delayed in such cases [10]. 

However, no case of NMOSD coexisting with compressive myelopathy due to OPLL has been reported so far. This is the first report to describe a case of cervical/thoracic OPLL and NMOSD with gait disturbance.

## Case presentation

History

A 65-year-old woman presented with a chief complaint of gait disorder. She was hospitalized in a previous hospital because of an infarction of the medulla oblongata and pons. She recovered after 10 days of hospitalization and was discharged. The following month, she became aware of stiffness and numbness in her right ankle, which gradually progressed to muscle weakness and sensory disturbance in both her lower extremities. Her medical history included hypertension, dyslipidemia, cerebral infarction, and Basedow's disease.

Clinical examination

On her first visit to our hospital, she was staggering even on flat ground with a spastic gait and her tandem gait was unstable. She had no motor deficits in the upper extremity (Manual Muscle Test (MMT): all 5). Her deep tendon reflexes of the lower extremities were exacerbated (patella tendon reflex ++, Achilles tendon reflex ++), and Babinski reflex was negative. Her sensory deficits included numbness and hypesthesia throughout both of her lower extremities. One month later, she became unable to stand. Her lower-extremity MMT was overall decreased with iliopsoas (IP), 3-/3; quadriceps (Q), 3/3; tibialis anterior (TA), 4/4; extensor hallucis longus (EHL),4/4; and gastrocnemius soleus (GS), 4/4. In the routine preoperative screening tests, there were no abnormal findings in the blood tests.

Imaging investigations

**Figure 1:**
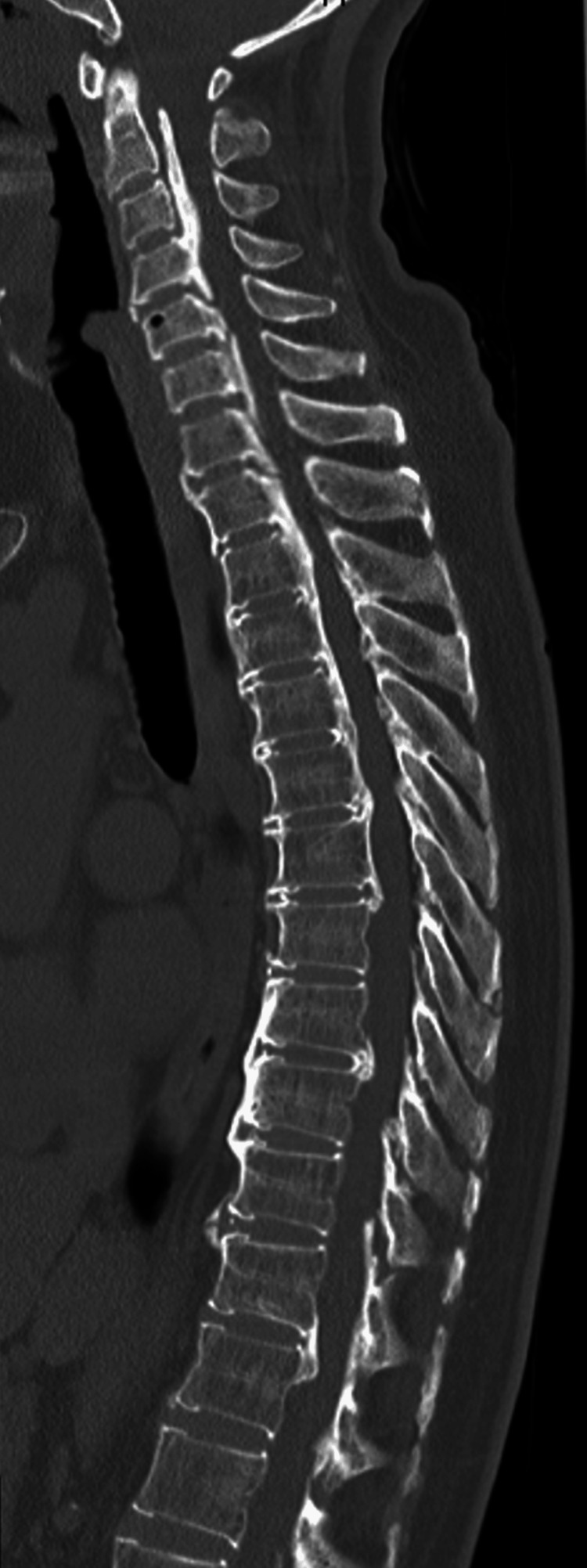
Computed tomography showed ossification of the posterior longitudinal ligament at C2-Th6, Th8/9, and Th11/12.

**Figure 2:**
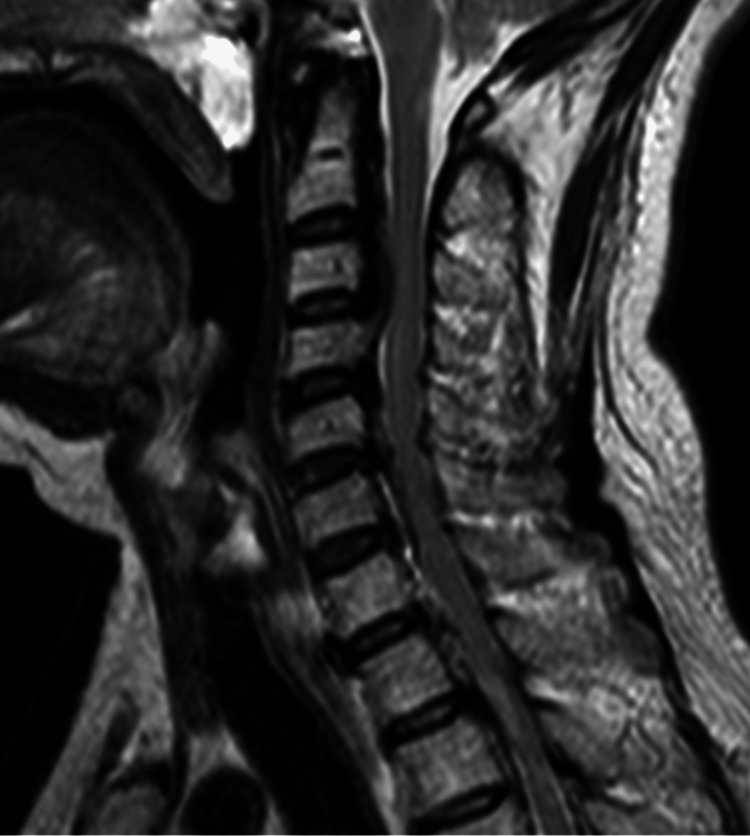
Cervical spine magnetic resonance imaging(MRI) showed spinal canal stenosis at C3 or lower and maximum stenosis at C6/7.

**Figure 3:**
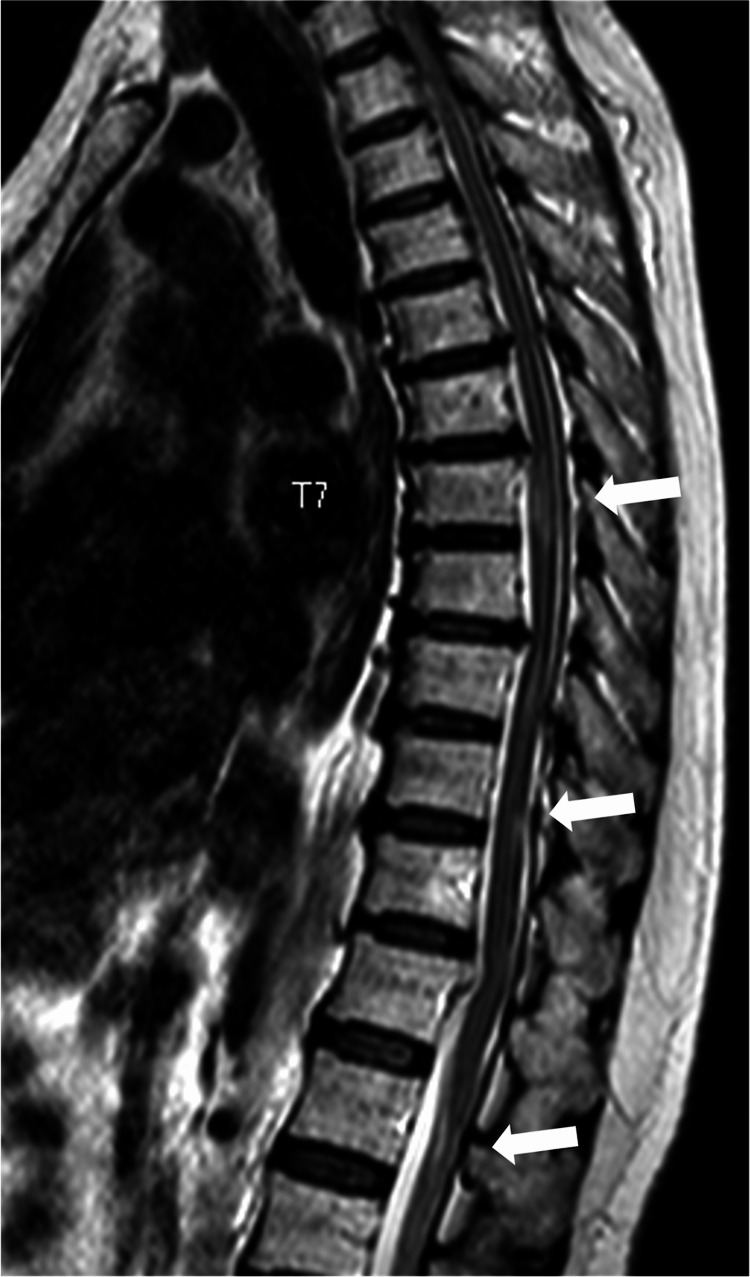
In the thoracic spine MRI, spinal canal stenosis was seen at Th5/6, Th6/7, and Th11/12. Intramedullary signal changes were seen in Th6, 7, 9, and 10/11(white arrow).

Computed tomography (CT) of her spine showed ossification of the posterior longitudinal ligament at C2-Th6, Th8/9, and Th11/12 (Figure 1), and magnetic resonance imaging (MRI) showed spinal canal stenosis at C3 or lower, and maximum stenosis at C6/7 (Figure 2). In the thoracic spine, spinal canal stenosis was seen at Th5/6, Th6/7, and Th11/12. Intramedullary signal changes were seen in Th6, 7, 9, and 10/11 (Figure 3). Her brain MRI showed no lesions characteristic of a demyelinating disease.

Procedures and treatment

Prior to surgery, we consulted neurologists because of intramedullary T2 hyperintensity in areas unrelated to the site of spinal cord compression and obtained an AQP4-antibody test following their suggestion. However, there were no symptoms other than progressive weakness in the lower limbs and sensory disturbances. Furthermore, because of the significant spinal cord compression caused by OPLL, the involvement of myelopathy due to OPLL could not be ruled out. Given the poor prognosis of progressive paralysis in compressive myelopathy, surgery was performed early.

**Figure 4:**
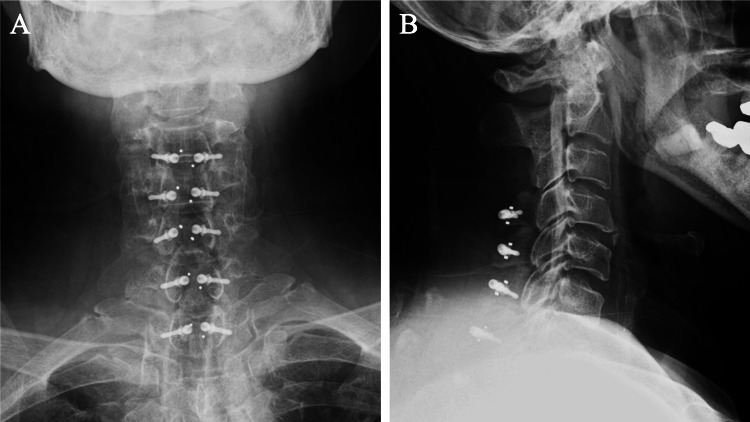
Postoperative radiograph of first operation (C2-Th2 decompression surgery, A: anteroposterior, B: lateral)

**Figure 5:**
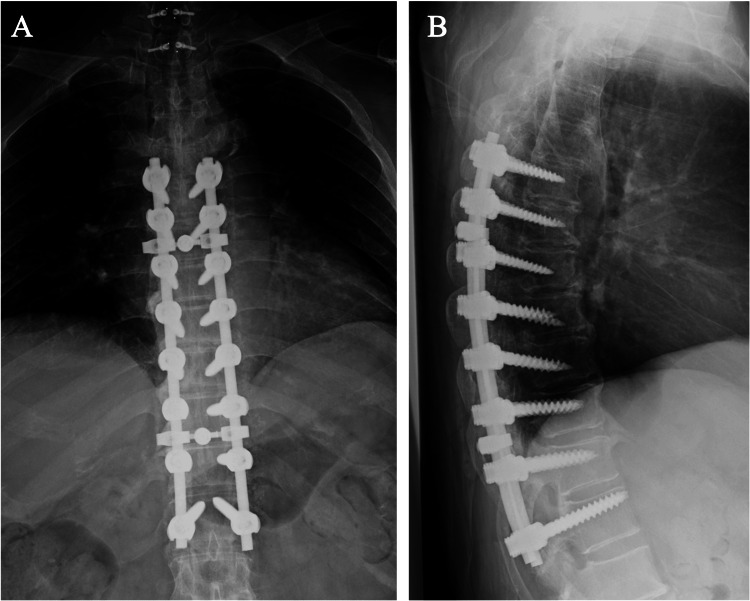
Postoperative radiograph of second surgery (Th5-Th12 decompression and fixation, A: anteroposterior, B: lateral)

The operation was performed in two stages. The first stage was a posterior decompression from C2 to Th2. A posterior approach was used to perform a laminectomy at C3, a double-door laminoplasty from C4 to Th1, and partial laminectomies at C2 and Th2 (Figure 4). One week later, the second stage of surgery was performed, which included posterior decompression and fixation from Th5 to Th12 (posterolateral fixation). Again, a posterior approach was used and the spinous processes were divided longitudinally from T5 to T10. Pedicle screws were inserted from T5 to T12, followed by a laminectomy from T5 to T12. Finally, autologous bone grafting was performed (Figure 5). There were no intraoperative complications and postoperative CT confirmed adequate bony decompression of the spinal cord and correct positioning of the pedicle screws.

Treatment outcome

However, after the first cervical and thoracic spine surgery, the muscle strength of the lower extremities gradually decreased. Just before the second surgery, the MMT level dropped to 1-2. Even after the second thoracic spine surgery, there was no improvement in muscle strength of the lower extremities. No spinal epidural hematoma was found on MRI.

Eight days after the second surgery, her AQP4-antibody were found to be positive, and she was diagnosed with NMOSD. Cerebrospinal fluid analysis showed no increase in cell count, but an increase in total protein concentration (312 mg/dL). There were no symptoms related to the optic nerve and the ophthalmological examination showed no abnormal findings.

After nine postoperative days, four series of steroid pulse treatments and six series of plasmapheresis treatments were performed. Thereafter, the muscle strength of the lower extremities gradually improved. Three months after the last operation, she was able to walk with a cane and was discharged home while taking oral steroids (prednisolone 20 mg/day) and an immunosuppressant (azathioprine 50 mg/day).

Summary of case presentation

A 65-year-old woman with cervicothoracic OPLL presented with progressive lower-extremity weakness and sensory loss. Due to the presence of intramedullary signal changes on MRI at sites inconsistent with the site of spinal cord compression, the possibility of demyelinating disease was also considered, leading to testing for AQP4 antibodies. However, due to the progressive nature of the lower limb symptoms and the consideration of OPLL as a contributing factor, early spinal surgery was performed. Postoperatively, there was a further decline in lower limb muscle strength and her AQP4 antibody was found to be positive, leading to a diagnosis of NMOSD. Treatment for NMOSD resulted in the recovery of lower limb muscle strength.

## Discussion

Spinal cord compression due to OPLL may present with progressive symptoms, in which case surgical treatment is urgent [1]. However, it has been reported that there was a significant delay in the diagnosis and treatment of symptomatic cervical spondylosis in patients with coexistent demyelinating disease [10]. The reason may be that clinical symptoms are similar, making it difficult to differentiate them based on symptoms alone.

Compressive myelopathy occasionally shows high-intensity lesions on T2-weighted images on MRI [11]. Although T2 high-intensity lesions are also seen in NMOSD, the signal change in NMOSD is characterized by long intramedullary changes over three or more complete vertebral segments, which are called longitudinally extensive transverse myelitis lesions (LETM) [12]. On the other hand, the absence of LETM in NMOSD is not uncommon [13], and diagnosis based on imaging findings alone is difficult. In this case, although a LETM lesion was not observed, intramedullary signal changes were seen in areas different from the compression site. This finding led us to perform the AQP4-antibody test before surgery, which was helpful for proper diagnosis. Therefore, when intramedullary signal changes are present in areas other than the area of spinal cord compression, or signal change extends over three vertebral bodies or more, it is worth considering testing AQP4-antibody for the possibility of NMOSD.

Complications of cervical spondylotic myelopathy and MS, which is a typical demyelinating disease, have been reported so far [10]. Although past literature recommended conservative treatment for CSM cases complicated by MS [14,15], more recent analyses have advocated for the effectiveness of surgery for CSM complicated by MS [10,16,17].

While combinations of CSM and MS have been reported, cases of NMOSD and OPLL have not been reported so far. Therefore, the efficacy and safety of spine surgery for patients with OPLL and NMOSD are unknown. In this case, early spinal decompression and fixation surgeries were performed confirming the evidence of spinal cord compression on imaging and facing progressive symptoms, in fear of poor neurological prognosis due to delayed surgery.

Despite the early surgical interventions for the cervical and thoracic spine, the patient’s muscle strength in the lower extremities temporarily decreased. It has been reported that increased body temperature may deteriorate neurological symptoms in MS and NMOSD (Uhthoff phenomenon) [18,19,20]. Since there was no direct injury of the spinal cord during surgeries, postoperative fever or inflammation may have caused a temporary exacerbation of neurological symptoms.

The lower-extremity weakness of the patient improved after drug therapy for NMOSD.

The primary reason for the improvement was thought to be that inflammation in the spinal cord was suppressed by the treatments. On the other hand, there was also the possibility that myelopathy has improved over time due to the spinal cord decompression effect of surgery. As a result, both treatments might have contributed to the improvement of symptoms.

It was not possible to state whether the surgical treatment was beneficial for cases with OPLL and NMOSD, as both diseases were treated almost simultaneously, which ultimately might lead to improvement of paralysis. Although the combination of OPLL and NMOSD is considered to be an extremely rare condition, it is necessary to accumulate and examine such case reports in the future.

## Conclusions

This report described a rare surgical case of cervical and thoracic OPLL with NMOSD for the first time. If there is a discrepancy between the site of severe stenosis and intramedullary signal changes on MRI, it is necessary to consider the possibility of coexisting inflammatory spinal pathology, such as NMOSD. It is not possible to evaluate the advantages and disadvantages of surgery in cases of combined OPLL and NMOSD based on this single case. Further collection and examination of case reports is crucial to enhance our understanding of these comorbidities and provide insights for future treatment development.

## References

[1] Boody BS, Lendner M, Vaccaro AR (2019). Ossification of the posterior longitudinal ligament in the cervical spine: a review. Int Orthop.

[2] Matsunaga S, Sakou T (2012). Ossification of the posterior longitudinal ligament of the cervical spine: etiology and natural history. Spine (Phila Pa 1976).

[3] Le HV, Wick JB, Van BW, Klineberg EO (2022). Ossification of the posterior longitudinal ligament: pathophysiology, diagnosis, and management. J Am Acad Orthop Surg.

[4] Yan L, Gao R, Liu Y, He B, Lv S, Hao D (2017). The pathogenesis of ossification of the posterior longitudinal ligament. Aging Dis.

[5] Stapleton CJ, Pham MH, Attenello FJ, Hsieh PC (2011). Ossification of the posterior longitudinal ligament: genetics and pathophysiology. Neurosurg Focus.

[6] Bakhsh W, Saleh A, Yokogawa N, Gruber J, Rubery PT, Mesfin A (2019). Cervical ossification of the posterior longitudinal ligament: a computed tomography-based epidemiological study of 2917 patients. Global Spine J.

[7] Carnero Contentti E, Correale J (2021). Neuromyelitis optica spectrum disorders: from pathophysiology to therapeutic strategies. J Neuroinflammation.

[8] Wingerchuk DM, Lennon VA, Lucchinetti CF (2007). The spectrum of neuromyelitis optica. Lancet Neurol.

[9] Tullman MJ (2013). Overview of the epidemiology, diagnosis, and disease progression associated with multiple sclerosis. Am J Manag Care.

[10] Youssef C, Barrie U, Elguindy M (2021). Compressive cervical myelopathy in patients with demyelinating disease of the central nervous system: improvement after surgery despite a late diagnosis. Cureus.

[11] McCormick JR, Sama AJ, Schiller NC, Butler AJ, Donnally CJ 3rd (2020). Cervical spondylotic myelopathy: a guide to diagnosis and management. J Am Board Fam Med.

[12] Wingerchuk DM, Banwell B, Bennett JL (2015). International consensus diagnostic criteria for neuromyelitis optica spectrum disorders. Neurology.

[13] Flanagan EP, Weinshenker BG, Krecke KN (2015). Short myelitis lesions in aquaporin-4-IgG-positive neuromyelitis optica spectrum disorders. JAMA Neurol.

[14] Meyer F, Sandovss G (1994). Unsuspected multiple sclerosis in patients with presumed chronic spondylotic myelopathy: report on 4 cases. Zentralbl Neurochir.

[15] Yerneni K, Nichols N, Burke JF, Traynelis VC, Tan LA (2019). Surgical management of patients with coexistent multiple sclerosis and cervical stenosis: a systematic review and meta-analysis. J Clin Neurosci.

[16] Lubelski D, Abdullah KG, Alvin MD (2014). Clinical outcomes following surgical management of coexistent cervical stenosis and multiple sclerosis: a cohort-controlled analysis. Spine J.

[17] Owiti W, Peev N, Arif S, Brady Z, AbdelHafiz T (2022). Is surgery beneficial for patients with concurrent multiple sclerosis and degenerative cervical myelopathy? A review of literature. Brain Spine.

[18] Park K, Tanaka K, Tanaka M (2014). Uhthoff's phenomenon in multiple sclerosis and neuromyelitis optica. Eur Neurol.

[19] Muto M, Mori M, Sato Y, Uzawa A, Masuda S, Uchida T, Kuwabara S (2015). Current symptomatology in multiple sclerosis and neuromyelitis optica. Eur J Neurol.

[20] Fraser CL, Davagnanam I, Radon M, Plant GT (2012). The time course and phenotype of Uhthoff phenomenon following optic neuritis. Mult Scler.

